# Shared decision-making in stroke: an evolving approach to improved patient care

**DOI:** 10.1136/svn-2017-000081

**Published:** 2017-04-16

**Authors:** Melissa J Armstrong

**Affiliations:** Department of Neurology, University of Florida College of Medicine, Gainesville, Florida, USA

**Keywords:** Shared decision making, decision making, stroke, values

## Abstract

Shared decision-making (SDM) occurs when patients, families and clinicians consider patients’ values and preferences alongside the best medical evidence and partner to make the best decision for a given patient in a specific scenario. SDM is increasingly promoted within Western contexts and is also being explored outside such settings, including in China. SDM and tools to promote SDM can improve patients’ knowledge/understanding, participation in the decision-making process, satisfaction and trust in the healthcare team. SDM has also proposed long-term benefits to patients, clinicians, organisations and healthcare systems. To successfully perform SDM, clinicians must know their patients’ values and goals and the evidence underlying different diagnostic and treatment options. This is relevant for decisions throughout stroke care, from thrombolysis to goals of care, diagnostic assessments, rehabilitation strategies, and secondary stroke prevention. Various physician, patient, family, cultural and system barriers to SDM exist. Strategies to overcome these barriers and facilitate SDM include clinician motivation, patient participation, adequate time and tools to support the process, such as decision aids. Although research about SDM in stroke care is lacking, decision aids are available for select decisions, such as anticoagulation for stroke prevention in atrial fibrillation. Future research is needed regarding both cultural aspects of successful SDM and application of SDM to stroke-specific contexts.

Shared decision-making (SDM) is an increasingly referenced and lauded approach to medical decision-making in Western countries, and its use is also spreading to other contexts including China^1^ and Malaysia.^2^ SDM is a partnership between patients (and families, where appropriate) and clinicians that considers patients’ values and preferences alongside medical evidence to make the best decisions for a given patient in a specific scenario.

In Western countries, arguments for SDM often focus on principles of autonomy and self-determination, particularly in the setting of clinical uncertainty. For many decisions, there is not a ‘right’ answer; SDM enables patients and families to choose the best option for them based on individual values, goals and considerations such as mechanism of administration, cost and side effect profile.

Arguments supporting the use of SDM go beyond these principles, however. Research suggests that SDM results in improved knowledge/understanding, satisfaction and trust,^3^ which are hoped to also lead to better health outcomes. Decision aids (DAs)—tools that guide patients, families and clinicians through the SDM process—increase knowledge, lower patients’ decisional conflict, reduce patient passivity in decision-making and the number of patients unable to decide, and result in more decisions for less-aggressive care.^4^ Research to date has focused more on these short-term outcomes of SDM rather than its long-term impact on health outcomes.^3 5 6^ A conceptual model of SDM, however, suggests that SDM can result in short-term, mid-term and long-range benefits for patients, clinicians/other healthcare professionals, organisations and healthcare systems, including improved decision-making, satisfaction, patient experiences, trust, health outcomes, cost-effectiveness and resource utilisation, along with decreases in litigation and professional burnout.^6^

## Approaches to SDM

SDM likely best occurs in the setting where a physician and a patient have an established relationship such that the physician knows the patient’s values and goals, informing how options are described and weighed during SDM.^7^ Even in the acute setting, such as the emergency department or during hospitalisation, understanding a patient’s background (eg, employment) and values prior to formal decision-making can provide important context for decisions.

There are multiple models for SDM with different numbers of outlined steps.^8–10^ When a clinical decision is needed, SDM starts by engaging patients and key supports in the process (table 1). This step requires the clinician to understand who the patient desires to participate in decision-making, such as a family member. In situations where a patient is incapacitated and unable to participate, SDM occurs with the surrogate decision maker. Even if a patient ultimately desires to defer the decision to a physician or family member, it is important to actively engage him/her in the SDM process.

**Table 1:** Steps to shared decision-making

Step 1	Engage patients (and other decision makers, if appropriate) in the decision-making process
Step 2	Describe the decision and the options available, including each option’s potential benefits and risks, how the options are different and what is unknown about the options (the uncertainty)
Step 3	Further assess the patient’s values and goals, specifically as they relate to the available options
Step 4	Make the decision together

Once patients and families are engaged, the second step (table 1) is specifically describing the decision and outlining the different options. In describing the different options, clinicians should use the available medical evidence to inform patients about the potential benefits and risks. It is also important to highlight when there is something unknown about the options (uncertainty) and to describe how the options are distinct. Differences between options include potential benefits and harms and considerations such as cost and invasiveness. This discussion should be individualised—for example, the balance of benefits and harms of clopidogrel for secondary stroke prevention will be different between a person with a prior history of bleeding gastric ulcers and a person with no such medical history.

Once the evidence is presented, the intersection between the options and the patient’s values and goals is explored (step 3, table 1). If a patient’s circumstances, values and goals were known prior to the initiation of SDM, the presentation of the options in step 2 should occur in that context. For example, if a patient lives alone and prioritises continued independence, the potential benefits and risks of each intervention are specifically presented with a reference to the likelihood of maintaining independence (eg, from successful treatment) or putting it at risk (eg, due to side effects). Regardless of prior knowledge of patient values, in step 3 a patient’s values as they specifically relate to the decision are explored. What is most important to the patient in this situation—expected functional recovery? Amount of risk? Cost?

Finally, a decision is made. Ideally, the patient makes the decision with the help of those friends or family members whom he or she has chosen for involvement. Sometimes patients prefer clinicians to make final decisions. In these circumstances, patients often still express a desire for participating in SDM,^11^ but request that the clinician select the best strategy after discussion. When the patient defers to clinicians, the burden is on the physician to target the decision to the patient’s stated values and goals, thus still using SDM to make the best individualised decision for that patient in that circumstance.

In many circumstances, particularly those encountered in the outpatient setting, re-evaluation is an important component of SDM. Anticipated ongoing benefits and risks may change based on the development of comorbidities; patients’ values and priorities may change based on their experiences with a medication or shifting life circumstances. In the outpatient clinic, for example, decisions regarding anticoagulation for secondary stroke prevention in the setting of atrial fibrillation should be reassessed over time. This is in contrast to certain acute stroke decisions, such as those regarding tissue plasminogen activator (t-PA), where the window for SDM is small, with little opportunity for re-evaluation.

### Values and goals

Within SDM, values tied clearly to diagnostic or therapeutic options such as efficacy, toxicity, quality of life, convenience and cost are often emphasised.^12^ Other values and goals may also inform patient decisions, however, and these can be critical to SDM.^13^ Global values reflect life priorities or beliefs, which may be religious or cultural in origin; these values impact all decisions. Global values can also represent value traits, such as risk aversion or a desire to try the ‘new’ thing, which also influences approaches to decision-making.^13^ External values reflect a patient’s choice to consider others’ values and preferences when making a decision.^13^ This occurs in Western cultures but may be more important in other cultures, such as in mainland China where family involvement in decision-making can reflect mutual benevolence and the Confucian ideal of family harmony,^14^ or places like Pakistan where the norm is family–doctor–patient triadic decision-making.^15^ Finally, situational values reflect context-specific factors that influence a decision differently now than in the past or future, such as an upcoming event (eg, a wedding) that may impact how long a patient is willing to remain in the hospital or rehabilitation.^13^

### The role of evidence-based medicine

Although SDM is often emphasised in discussions of personalised and patient-centred care, it is critical to note that this process relies on evidence-based medicine. Evidence-based medicine is foundational to step 2 of SDM (table 1), where patients, families and clinicians discuss the evidence (or uncertainty/lack of evidence) of benefits and harms for each potential option. It is only by knowing the available evidence that patients and families can make informed decisions. To present this evidence, clinicians can reference original research or use tools such as DAs, systematic reviews or evidence-based guidelines, each of which summarises known evidence in response to a specific question or choice.

## SDM and stroke

Most recent publications on SDM in stroke care focus on oral anticoagulation for stroke prevention in atrial fibrillation.^16–20^ This is a decision where SDM clearly plays an important role given differences in individual risks based on comorbidities, multiple options with different potential benefits, risks, costs and time requirements (eg, for international normalized ratio [INR] monitoring), and obvious value assessments relating to potential outcomes such as stroke and bleeding.

Less research exists for other decisions relating to stroke, and currently available DAs may not meet decision aid standards.^21^ A 2013 review of patient tools designed for decision-making regarding thrombolytic treatment identified that available tools lacked key development stages, presented outcome probabilities poorly and failed to completely describe potential benefits and risks.^22^ Subsequently, the COMPuterized decsion Aid for Stroke thrombolysiS (COMPASS) tool, a computerised DA for thrombolysis in acute stroke, was developed with clinicians, patients, families and modelling techniques. Using the tool took a median time of only 2.8 min in early pilot testing, but additional study is required.^23 24^ DAs have particular potential for improving care in this emergent setting, where SDM is challenged by the time limitations for effective thrombolysis, the need to engage patients and families and convey knowledge in the context of the shock and effects of an acute stroke, and the need to incorporate personal values into a decision that relies heavily on physician expertise.^25^

### Barriers to SDM

Research on barriers to SDM is largely conducted in Western contexts. Identified barriers to SDM include physician and patient attitudes towards SDM,^26 27^ lack of familiarity and experience with SDM,^26^ lack of continuity of care,^27^ physician knowledge regarding evidence,^26 27^ the physician–patient relationship,^27^ insufficient explanations,^27^ use of medical terminology,^27^ the ability of patients and families to understand and use health-related information (health literacy),^27 28^ lack of resources^26 27^ and time.^10 26 27^

Research regarding SDM in China is extremely limited, but identified barriers overlap with those described elsewhere and include lack of resources, time, physician communication skills, patient–physician relationships, the health literacy of patients and families, and unrealistic patient and family expectations.^1^ Despite these barriers, a recent study found it feasible to implement the use of a statin DA for cardiovascular risk reduction in two teaching hospitals in Northern China.^29^ Additional barriers identified in this study included lack of privacy for uninterrupted discussions, family dominance within some encounters, lack of applicability of data within Western DAs to Chinese contexts, and low health literacy requiring additional cardiovascular education in order for patients to effectively use the tool.^29^

### Facilitators of SDM

The most commonly described facilitators of SDM are clinician-related: clinician motivation and the perception that SDM improves the clinical process and patient outcomes.^26^ Patient-identified facilitators include continuity of care, good relationships between patients and clinicians, trust, adequate time, engagement of various members of the healthcare team (eg, nurses, in addition to doctors), a sense of partnership, encouragement of patients to participate and ask questions, the provision of sufficient information, use of plain language, and patient engagement and ownership in the process.^27^

DAs are practical facilitators of SDM, although they are insufficient on their own and have some limitations.^27^ DAs are useful for addressing barriers to SDM, such as lack of familiarity with SDM, physician knowledge regarding evidence, and provision of sufficient and understandable information, as DAs walk clinicians, patients and families through the SDM process and describe the medical evidence in plain language, often using visual aids.

Although there are few published stroke-related DAs, approaches exist for helping clinicians develop tools for commonly encountered decisions, such as Option Grids.^30^ In the absence of formal tools, other patient education materials can be helpful in promoting step 2 of SDM, such as those available through neurology and stroke organisations. Ideal tools will be culturally and context-sensitive, something of particular importance as SDM spreads to non-Western contexts.^1 29^

## Conclusions

SDM is an increasingly promoted approach for patients, families and clinicians to partner to make the best medical decisions for each individual in a particular moment by using the best medical evidence. Although long-term benefits for patients, families, clinicians, hospitals and health systems have yet to be explored, SDM has known benefits on decision-making and satisfaction and has the potential for improving other outcomes as well. Every decision within stroke care has potential for improvement with SDM, whether relating to thrombolysis, goals of care, diagnostic assessments, rehabilitation strategies or secondary stroke prevention. Although SDM is necessarily context-specific, development of DAs for commonly faced decisions within vascular neurology may improve stroke care. Future research is needed regarding the cultural elements of SDM in general and also within the field of stroke.

## References

[1] Huang R, Gionfriddo MR, Zhang L, et al Shared decision-making in the people’s Republic of China: current status and future directions. Patient Prefer Adherence 2015;9:1129–41. doi:10.2147/PPA.S82110 26273201 10.2147/PPA.S82110 PMC4532212

[2] Ng CJ, Lee PY, Lee YK, et al An overview of patient involvement in healthcare decision-making: a situational analysis of the Malaysian context. BMC Health Serv Res 2013;13:408 doi:10.1186/1472-6963-13-408 24119237 10.1186/1472-6963-13-408 PMC3852442

[3] Shay LA, Lafata JE Where is the evidence? A systematic review of shared decision making and patient outcomes. Med Decis Making 2015;35:114–31. doi:10.1177/0272989X14551638 25351843 10.1177/0272989X14551638 PMC4270851

[4] Stacey D, Légaré F, Col NF, et al Decision aids for people facing health treatment or screening decisions. 2014;1:CD001431. doi:10.1002/14651858.CD001431.pub3 10.1002/14651858.CD001431.pub4 24470076

[5] Rathert C, Wyrwich MD, Boren SA Patient-centered care and outcomes: a systematic review of the literature. Med Care Res Rev 2013;70:351–79. doi:10.1177/1077558712465774 23169897 10.1177/1077558712465774

[6] Elwyn G, Frosch DL, Kobrin S Implementing shared decision-making: consider all the consequences. Implement Sci 2016;11:114 doi:10.1186/s13012-016-0480-9 27502770 10.1186/s13012-016-0480-9 PMC4977650

[7] Armstrong MJ, Shulman LM, Vandigo J, et al Patient engagement and shared decision-making: what do they look like in neurology practice? Neurol Clin Pract 2016;6:190–7. doi:10.1212/CPJ.0000000000000240 27104070 10.1212/CPJ.0000000000000240 PMC4828680

[8] Elwyn G, Frosch D, Thomson R, et al Shared decision making: a model for clinical practice. J Gen Intern Med 2012;27:1361–7. doi:10.1007/s11606-012-2077-6 22618581 10.1007/s11606-012-2077-6 PMC3445676

[9] AHRQ. The SHARE approach. Essential steps of shared decision making: quick reference guide (Workshop curriculum: tool 1). AHRQ pub no 14-0034-1-EF: april 2014. 2017;3 https://www.ahrq.gov/professionals/education/curriculum-tools/shareddecisionmaking/workshop/index.html.

[10] Légaré F, Witteman HO Shared decision making: examining key elements and barriers to adoption into routine clinical practice. Health Aff (Millwood) 2013;32:276–84. doi:10.1377/hlthaff.2012.1078 23381520 10.1377/hlthaff.2012.1078

[11] Levinson W, Kao A, Kuby A, et al Not all patients want to participate in decision making. A national study of public preferences. J Gen Intern Med 2005;20:531–5. doi:10.1111/j.1525-1497.2005.04101.x 15987329 10.1111/j.1525-1497.2005.04101.x PMC1490136

[12] Schnipper LE, Davidson NE, Wollins DS, et al American Society of Clinical Oncology statement: a conceptual framework to assess the value of cancer treatment options. J Clin Oncol 2015;33:2563–77. doi:10.1200/JCO.2015.61.6706 26101248 10.1200/JCO.2015.61.6706 PMC5015427

[13] Armstrong MJ, Mullins CD Value assessment at the point of care: incorporating patient values throughout care delivery and a draft taxonomy of patient values. Value Health 2017;20:292–5. doi:10.1016/j.jval.2016.11.008 28237212 10.1016/j.jval.2016.11.008 PMC5417067

[14] Rui D A family-oriented decision-making model for human research in mainland China. J Med Philos 2015;40:400–17. doi:10.1093/jmp/jhv013 26142439 10.1093/jmp/jhv013

[15] Aslam F, Aftab O, Janjua NZ Medical decision making: the family-doctor-patient triad. PLoS Med 2005;2:e129 doi:10.1371/journal.pmed.0020129 15971934 10.1371/journal.pmed.0020129 PMC1160566

[16] Kaiser K, Cheng WY, Jensen S, et al Development of a shared decision-making tool to assist patients and clinicians with decisions on oral anticoagulant treatment for atrial fibrillation. Curr Med Res Opin 2015;31:2261–72. doi:10.1185/03007995.2015.1096767 26390360 10.1185/03007995.2015.1096767

[17] Eckman MH, Wise RE, Naylor K, et al Developing an atrial fibrillation guideline support tool (AFGuST) for shared decision making. Curr Med Res Opin 2015;31:603–14. doi:10.1185/03007995.2015.1019608 25690491 10.1185/03007995.2015.1019608 PMC4708062

[18] Ferguson C, Hendriks J Partnering with patients in shared decision-making for stroke prevention in atrial fibrillation. Eur J Cardiovasc Nurs 2017;16:178–80. doi:10.1177/1474515116685193 28240139 10.1177/1474515116685193

[19] Seaburg L, Hess EP, Coylewright M, et al Shared decision making in atrial fibrillation: where we are and where we should be going. Circulation 2014;129:704–10. doi:10.1161/CIRCULATIONAHA.113.004498 24515956 10.1161/CIRCULATIONAHA.113.004498

[20] Pandya E, Bajorek BV Assessment of Web-based education resources informing patients about stroke prevention in atrial fibrillation. J Clin Pharm Ther 2016;41:667–76. doi:10.1111/jcpt.12446 27704588 10.1111/jcpt.12446

[21] Elwyn G, O’Connor A, Stacey D, et al International Patient Decision Aids Standards (IPDAS) Collaboration. Developing a quality criteria framework for patient decision aids: online international delphi consensus process. BMJ 2006;333:417 doi:10.1136/bmj.38926.629329.AE 16908462 10.1136/bmj.38926.629329.AE PMC1553508

[22] Flynn D, Ford GA, Stobbart L, et al A review of decision support, risk communication and patient information tools for thrombolytic treatment in acute stroke: lessons for tool developers. BMC Health Serv Res 2013;13:225 doi:10.1186/1472-6963-13-225 23777368 10.1186/1472-6963-13-225 PMC3734197

[23] McMeekin P, Flynn D, Ford GA, et al Development of a decision analytic model to support decision making and risk communication about thrombolytic treatment. BMC Med Inform Decis Mak 2015;15:90 doi:10.1186/s12911-015-0213-z 26560132 10.1186/s12911-015-0213-z PMC4642673

[24] Flynn D, Nesbitt DJ, Ford GA, et al Development of a computerised decision aid for thrombolysis in acute stroke care. BMC Med Inform Decis Mak 2015;15:6 doi:10.1186/s12911-014-0127-1 25889696 10.1186/s12911-014-0127-1 PMC4326413

[25] Murtagh MJ, Burges Watson DL, Jenkings KN, et al Situationally-sensitive knowledge translation and relational decision making in hyperacute stroke: a qualitative study. PLoS One 2012;7:e37066 doi:10.1371/journal.pone.0037066 22675477 10.1371/journal.pone.0037066 PMC3365903

[26] Légaré F, Ratté S, Gravel K, et al Barriers and facilitators to implementing shared decision-making in clinical practice: update of a systematic review of health professionals’ perceptions. Patient Educ Couns 2008;73:526–35. doi:10.1016/j.pec.2008.07.018 18752915 10.1016/j.pec.2008.07.018

[27] Joseph-Williams N, Elwyn G, Edwards A Knowledge is not power for patients: a systematic review and thematic synthesis of patient-reported barriers and facilitators to shared decision making. Patient Educ Couns 2014;94:291–309. doi:10.1016/j.pec.2013.10.031 24305642 10.1016/j.pec.2013.10.031

[28] Edwards M, Davies M, Edwards A What are the external influences on information exchange and shared decision-making in healthcare consultations: a meta-synthesis of the literature. Patient Educ Couns 2009;75:37–52. doi:10.1016/j.pec.2008.09.025 19036550 10.1016/j.pec.2008.09.025

[29] Huang R, Song X, Wu J, et al Assessing the feasibility and quality of shared decision making in China: evaluating a clinical encounter intervention for chinese patients. Patient Prefer Adherence 2016;10:2341–50. doi:10.2147/PPA.S115115 27881912 10.2147/PPA.S115115 PMC5115624

[30] Elwyn G, Lloyd A, Joseph-Williams N, et al Option Grids: shared decision making made easier. Patient Educ Couns 2013;90:207–12. doi:10.1016/j.pec.2012.06.036 22854227 10.1016/j.pec.2012.06.036

